# Geographical approach analysis of the impact of air pollution on newborn intrauterine growth and cord blood DNA damage in Mexico City

**DOI:** 10.1038/s41370-023-00618-x

**Published:** 2023-12-12

**Authors:** Jorge A. Maciel-Ruiz, Nancy Reynoso-Noverón, David A. Rodríguez-Moreno, Pavel Petrosyan, Jorge H. Limón-Pacheco, Andrés E. Nepomuceno-Hernández, Rodrigo Ayala-Yañez, Rogelio Robles-Morales, Citlalli Osorio-Yáñez, Claudia María García-Cuellar, María E. Gonsebatt

**Affiliations:** 1 Laboratorio de Carcinogénesis y Medio Ambiente, Subdirección de Investigación Básica, Instituto Nacional de Cancerología, Ciudad de México, México; 2 Centro de Investigación en Prevención, Instituto Nacional de Cancerología-Universidad Nacional Autónoma de México, México City, México; 3 Departamento de Medicina Genómica y Toxicología Ambiental, Instituto de Investigaciones Biomédicas, Universidad Nacional Autonóma de México, Ciudad de México, México; 4 Departamento de Biología Celular, Centro de Investigación y Estudios Avanzados del Instituto Politécnico Nacional, Ciudad de México, México; 5 Centro de Investigación Materno Infantil del Grupo de Estudios al Nacimiento, Asociación Hispano Mexicana, Ciudad de México, México; 6 División de Investigación de la Unidad Médica de Alta Especialidad, Hospital de Gineco-Obstetricia 3 “Dr. Víctor Manuel Espinosa de los Reyes Sánchez”, Centro Médico Nacional “La Raza”, Instituto Mexicano del Seguro Social, Ciudad de México, México; 7 Laboratorio de Fisiología y Transplante Renal, Unidad de Investigación en Medicina Traslacional, Instituto de Investigaciones Biomédicas, Universidad Nacional Autónoma de México and Instituto Nacional de Cardiología Ignacio Chávez, México City, 14080 México

**Keywords:** particulate matter, DNA adducts, Mexico City, emission sources, birth weight and length, gestational age

## Abstract

**Background:**

Few epidemiologic studies have focused on the specific source of ambient air pollution and adverse health effects in early life. Here, we investigated whether air pollutants from different emission sources were associated with decreased birth anthropometry parameters and increased DNA adduct formation in mother-child pairs residing in the Mexico City Metropolitan Area (MCMA).

**Methods:**

This cross-sectional study included 190 pregnant women recruited during their last trimester of pregnancy from two hospitals at MCMA, and a Modeling Emissions Inventory (MEI) to calculate exposure to ambient air pollutants from different emissions sources (area, point, mobile, and natural) for two geographical buffers 250 and 750 m radii around the participants households.

**Results:**

Contaminants were positively correlated with umbilical cord blood (UCB) adducts, but not with maternal blood (MB) adducts. PM10 emissions (area and point sources, overall emissions), PM2.5 (point sources), volatile organic compounds (VOC), total organic compounds (TOC) from point sources were positively correlated with UCB adducts. Air pollutants emitted from natural sources were correlated with a decrease in MB and UCB adducts. PM10 and PM2.5 were correlated (*p* < 0.05) with a decrease in birth weight (BW), birth length (BL) and gestational age at term (GA). In multivariate analyses adjusted for potential confounders, PM10 was associated with an increase in UCB adducts. PM10 and PM2.5 from overall emissions were associated with a decrease in BW, BL and GA at term.

**Impact:**

- Results suggested higher susceptibility of newborns compared to mothers to damage related to ambient air pollution. PMs are associated with birth anthropometry parameters and DNA damage in adjusted models, highlighting the need for more strict regulation of PM emissions.

## Introduction

Ambient air pollution is a complex mixture of chemicals, including particles, vapors and gases emitted from natural or anthropogenic sources [1, 2]. Particulate matter (PM) emissions come from natural sources (soil erosion, pollen, forest fires, and volcanic eruptions) and anthropogenic activities such as burning fossil fuels in households, industrial processes, and mobile sources [3, 4]. Natural and anthropogenic sources also deliver organic chemicals into the atmosphere, among them, volatile organic compounds (VOC) are chemicals that exist as gases or vapors at room temperature; VOC are emitted from numerous products and activities, including paints, solvents, tools, clothes, cleaning, and cooking, among others [5–7]. Some VOC examples are benzene, toluene, and xylene, which are part of the TOC (total organic compounds) and TOX (toxic compounds) emissions inventories reported biannually by the SEDEMA (Mexico City’s Secretariat of the Environment) in the Mexico City Metropolitan Area (MCMA) [8, 9]. The densely populated MCMA has severe air pollution problems due to the coexistence of large industrial and commercial establishments, circulating vehicles, and reduced distribution of green areas per inhabitant in the highest impact zones [8–11].

Prenatal exposure to environmental air pollutants is associated with adverse pregnancy and neonatal outcomes with potential clinical implications. For example, prenatal exposure to polycyclic aromatic hydrocarbons (PAH) and PM has been associated with an increased risk of intrauterine growth restriction (IUGR) [12, 13], preterm delivery [14, 15], low birth weight [16, 17], congenital anomalies [18, 19], and other adverse outcomes at delivery [20, 21]. Exposure to VOC is a risk factor for adverse health effects such as low birth weight, asthma, and leukemia [22, 23]. The International Agency for Research on Cancer (IARC) has classified outdoor air pollution as a Group 1 carcinogen [4]. In addition, exposure to air pollution is linked to different types of childhood cancer, especially leukemia [24–27]. PAH-DNA adducts are stablished biomarkers of cancer risk [28]. PAH-DNA adducts in newborns are indicators of transplacental genotoxic effects of air pollutants [29–31]. PAHs are present in VOC, TOX, TOC emissions and are also part of PM composition [6–9, 32]. PAHs metabolism by CYP enzymes produces electrophilic PAH metabolites that can bind to DNA nitrogenous bases forming DNA-adducts [28, 33]. Alternatively, PAHs can be also converted to PAHs semiquinone radicals with reactive oxygen species (ROS) production. ROS production can trigger multiple cellular signaling pathways not directly related to mutagenic and cytotoxic effects such as NFkb, SAPK/JNK y p38 [33]. Recently, Tavella et al. 2022 found that a greater risk of DNA damage was associated with birth weight and the presence of respiratory diseases in newborns admitted to neonatal intensive care unit [34]. Similarly, Da Correggio et al. (2021) reported that the frequency of micronuclei was associated with inadequate birth weight in newborns from Brazil [35]. We previously showed a relationship between prenatal exposure to PM and a significant increase in maternal and cord blood DNA adducts in the MCMA area [11]. However, a gap in the literature is the need for studies assessing the relationship between ambient air pollution levels from different emission sources, DNA adducts, and birth anthropometry. This study will provide a better understanding of the source of PM and VOC emissions related to anthropometry at birth and DNA adducts considering emission sources near the residence of the study participants rather than the total concentrations in the zone. The advantage of considering the emission sources of contaminants is that provides a narrower definition of pollutant chemical nature and may identify sources of contaminants with harmful health effects [36].

This study aimed to assess the relationship between PM, VOC, TOC and TOX emissions from different sources and birth weight (BW), birth length (BL), gestational age (GA), and DNA adducts in both mothers and newborns residing in MCMA.

## Materials and Methods

### Study subjects and their characterization

Two hundred fifty-eight pregnant women participated in this study and signed an informed consent form. Volunteers were recruited during third trimester of pregnancy from two public hospitals: 150 pregnant women from La Raza Hospital, Alta Especialidad en Ginecología y Obstetricia No. 3 Hospital, in the Center-North zone of Mexico City [11] and 108 participants from the maternity hospital of the Centro de Investigación Materno Infantil del Grupo de Estudios del Nacimiento (CIMIGen, for its Spanish initials) located in the Eastern zone of Mexico City. This study was approved by the Ethics Committees of the UNAM, Instituto de Investigaciones Biomédicas, IMSS (Instituto Mexicano del Seguro Social), and CIMIGen. Clinical information was obtained from maternity health records. Additionally, we applied questionnaires to collect information on occupation, drug use, exposure to genotoxic agents, nutritional habits, smoking and socioeconomic status. Questionnaires were coded. Information was analyzed blind. The inclusion criteria were as follows: non-smokers, 19–35 years old, residing in the MCMA in the last trimester of pregnancy, no chronic diseases (such as hypertension, asthma, gestational diabetes, or cancer), not occupationally exposed to PAH, and no pregnancy complications such as premature rupture of membranes. All women received iron and folic acid supplements during their entire pregnancy as part of their prenatal care. Blood and urine samples were collected at delivery. All pregnancies were at term, and we did not exclude multiple pregnancies. PAH-DNA adducts measurements in maternal (MB adducts) and umbilical cord blood (UCB adducts) were performed using the ^32^P postlabelling assay. DNA adducts were measured only when both MB and UCB blood samples provided an adequate DNA concentration. CMR ratio was calculated as UCB/MB adducts relationship. An increase in CMR ratio indicates more DNA damage in the newborn and a decrease in CMR more DNA damage in the mother. Urinary cotinine was measured employing One Step Cotinine Test Device (Certum Diagnostics, Monterrey, N.L. México). The threshold value for cotinine in urine was ≥200 ng/ml. Trained nurses or physicians obtained APGAR scores, gestational age (GA), birth weight (BW) and birth length (BL) parameters and information were added to their newborns ´vital records [11].

### Exposure assessment

According to the Secretariat of Environment and Natural Resources of Mexico (SEMARNAT), TOX are gases or particles suspended in the air, which can have long or short-term effects on human health, heavy metals included. This category englobes a list of 189 pollutants and can be consulted at the Emission Inventory website (http://www.aire.cdmx.gob.mx). TOC are a set of gases that include carbon compounds except for carbonic acid, carbonates, metal carbides, CO, and carbon dioxide. Supplementary Table 1 shows the list of VOC, TOC and TOX reported by SEDEMA [8, 9].

We assigned exposure to PM10, PM2.5, VOC, TOX, and TOC from four different emission sources in 1 km x 1 km grid using two geographic buffers, 250 and 750 m around households of each study participant. The first step in the exposure assignment was to obtain the geographical coordinates of the residence address of study participants using Google My Maps (http://www.google.com/mymaps). Then, we loaded geographical coordinates into a geographic information system (GIS) using ArcGIS Pro software (Environmental System Research Institute, USA) in combination with the data grid from the Modeling Emissions Inventory (MEI) database. According to computational and geographic modelling, the MEI data catalog provides emission levels of several pollutants (reported in tons per year). The MEI provides 1 × 1 km grids allowing high-resolution analysis of the annual average concentrations of atmospheric pollutants for each 1 km2 quadrant of pollutant and emission source [8, 9, 36]. We used 250 or 750 m radii around each household to calculate annual medians for PM10, PM2.5, VOC, TOX, and TOC from different emission sources. We chose these two radii because previous epidemiologic studies on birth anthropometry or diseases related to DNA damage included these radii within the range of distance from households to exposure sources: 250 m [17, 24, 37] y 750 m radius [18]. Additionally, we included the data of all the quadrants of the modelling grid in calculating the corresponding median if two or more quadrants were in the 250 or 750 radii of the participant household.

Only annual values of contaminants were available from SEDEMA website. We used the 2014 MEI of the Mexico City Environment Secretariat (SEDEMA, for its Spanish initials), available on the SEDEMA official website at http://www.aire.cdmx.gob.mx/. We decided to use the 2014 MEI to calculate annual exposure to contaminants for births occurring during 2014, 2015, and 2016 because most emission sources did not suffer significant variations over time (2014 vs. 2016) and MEI for 2015 was unavailable [8, 9].

We considered the following emission sources:

i. Area sources include domestic, fuel, solvent, waste, agricultural, livestock emissions, and small businesses and services.
ii. Point sources are represented by fixed-location industrial factories, businesses, and service facilities that generate polluting emissions into the atmosphere through regulated industrial, commercial, or service operations or processes.
iii. Mobile sources are represented by automotive transport that circulates through the MCMA roads.
iv. Natural sources generate emissions by biological processes in vegetation (trees) and soils, biogenic emissions (VOC from vegetation and NOx nitrogen oxides from the ground), and wind erosion [8].

### Statistical analysis

We performed exploratory analyses to assess data quality and consistency. Shapiro-Wilk test was employed to check the normality of the continuous variables. Ambient air pollutants and UCB and MB adducts were not normally distributed. Continuous variables were described as mean and standard deviation (SD) or median and interquartile range (IQR). Frequencies and percentages were reported for categorical variables. Wilcoxon paired-sample test was used to assess differences between MB and UCB adduct levels. We used Spearman’s correlations because of non-parametric distribution of the exposures and DNA adducts to examine: (1) the relationship between MB and UCB adduct levels; (2) correlations between air pollutant emissions from different sources and MB and UCB adduct levels; 3) correlations between air pollutants and GA, BW, and BL.

We analyzed associations between exposures (one contaminant at a time) and outcomes (birth anthropometry or DNA adducts) using multiple regression analyses adjusted for potential confounders. We used the forward stepwise method to select confounders and we include variables that impact exposure-outcome association, modify the R2 of the model (>10%) or those previously associated with the outcome or the exposure according to the literature [15, 16]. Exposures were analyzed one at a time because strong correlations existed between any two groups of contaminants increasing the VIF (variance inflation factor) of the model more than 4. All our models were adjusted for maternal occupation, maternal age, parity, and exposure to environmental tobacco smoke. In multiple regression analyses, ambient air pollutants and UCB adducts levels were log transformed to approximate normal distributions. Thus, we interpreted the coefficient as the percentage increase in the dependent variable (UCB adducts) per 1% increase in the independent variable (PM exposure). In multiple linear regression models, birth anthropometry parameters or gestational age were not log-transformed. Therefore, per 1% increase in the independent variable (PM exposure) the dependent variable (BW or BL or GA) increases or decreases by (coefficient/100) units [38].

Statistical analysis was performed using GraphPad Prism 9.0, and a *P*-Value < 0.05 was considered statistically significant.

## Results

### Study population

Residential address information was available for 190 of the 258 participants who met the inclusion criteria. We were not able to georeferencing 26.4% (68 out of 258) of the address provided by the participants because households were in streets or neighborhoods that municipal authorities or Google Maps could not validate; this, probably related to the accelerated urban growth in the MCMA. For DNA adducts, the ^32^P radioactive test was performed in 136 pairs. Thus, we have a total of 190 participants with information on air pollution exposure and birth anthropometry and 136 participants with information on air pollution exposure and DNA adducts. The average maternal age was 28.1 ± 5.1 years, and the average parity was 1.9 ± 1.1. 52.1% of women were employed and 47.4% unemployed (Table 1). 49% reported living with at least one smoker. None was positive for urinary cotinine, indicating no active tobacco smoke exposure in our population.

**Table 1:** Demographic characteristics and biomarkers of the study population.

Participants with georeferenced location (*n*)	190
**Maternal Characteristics**	
Age (years, mean ± SD)	28.1 ± 5.1
Parity (previous pregnancies, mean ± SD)	1.9 ± 1.1
Occupation, *n* (%)	
Housewife	90 (47.4%)
Employed	99 (52.1%)
Non reported	1 (0.5%)
Self-reported secondhand smoke exposure, *n* (%)	
Yes	93 (49%)
No	95 (50%)
Non reported	2 (1%)
Positive urine cotinine test for active tobacco smoke exposure	None
**Neonate anthropometry**	
Gestational age (weeks, mean ± SD)	38.9 ± 1.2
Weight (grams, mean ± SD)	3127 ± 439.4
Length (centimeters, mean ± SD)	49.9 ± 1.2
APGAR 5 min (mean ± SD)	8.9 ± 0.3
Sex, *n* (%)	
Female	92 (48.42%)
Male	95 (50.0%)
Non reported	3 (2%)
**Biomarkers (*n*)**	136
PAH-DNA adducts/10^8^ nucleotides	
Maternal blood (median ± IQR)	0.98 [0.44, 1.54]
Cord Blood (median ± IQR)	1.02^a^ [0.58, 1.89]
Cord blood/Maternal blood adduct Ratio (CMR), *n* (%)	
CMR > 1	82 (60.30%)
CMR ≤ 1	54 (39.70%)
Spearman correlation (rho coefficient, *p*-value)	
Maternal vs Cord Blood adducts	0.56 < 0.0001

*SD* Standard Deviation, *IQR* Interquartile Range.

^a^Non-Parametric Wilcoxon Paired Test: *p* = 0.0028.

The average GA of the newborns was 38.9 ± 1.2 weeks, the mean value for BW was 3127 ± 439.4 grams, and the BL was 49.9 ± 1.2 cm. 48.4% and 50% of newborns were females and males, respectively. The mean value of APGAR score was 8.9 ± 0.3. The median values for MB and UCB adducts per 10^8^ nucleotides were 0.98 and 1.02, respectively. UCB adducts were statistically significantly higher than MB adducts (Table 1). We found a significant correlation between MB and UCB adduct levels (*r* = 0.56; *p* < 0.0001). UCB/MB adduct ratio (CMR) values above 1 were obtained in 60.3% of the cases (82 pairs out of 136; Table 1).

### Air pollution exposure and distribution

Table 2 shows PM2.5, PM10, VOC, TOC and TOX concentrations for each emission source in the 250 and 750 radii. All air pollutants from point sources were statistically significantly higher in the 750 m radius than in the 250 m radius. PM10, VOC, and TOC emissions from natural sources were higher, although they did not reach statistical significance (p ∼0.07) in the 750 m radius (vs 250 m radius). Figure 1 shows the areal distribution of the total annual emission of PM10, PM2.5 and VOC. TOX and TOC spatial distributions were like VOC.

**Table 2:** Exposure emissions of air pollutants estimated using two geographical buffers: 750 or 250 m radii around volunteers’ households (Data expressed in tons per year).

	Buffers used to estimate the level of exposure (radius)				
Pollutant and source	750 m		250 m		
	Median	IQR	Median	IQR	*p-*value
**PM10**					
Area sources	5.28	[3.11, 8.61]	4.98	[2.97, 8.56]	0.491
Point sources	0.14	[0.01, 1.69]	0.01	[0.00, 0.49]	<0.000
Mobile sources	2.50	[1.37, 4.86]	2.37	[1.34, 4.87]	0.679
Natural sources	0.03	[0.01, 0.05]	0.02	[0.01, 0.04]	0.070#
Overall emissions	10.60	[5.76, 16.06]	9.15	[4.90, 15.93]	0.282
**PM2.5**					
Area sources	2.89	[2.22, 3.76]	2.86	[2.17, 3.95]	0.887
Point sources	0.08	[0.00, 1.05]	0.01	[0.00, 0.27]	<0.000
Mobile sources	1.28	[0.610, 2.61]	1.16	[0.60, 2.64]	0.548
Natural sources	0.01	[0.00, 0.01]	0.01	[0.00, 0.01]	0.080
Overall emissions	5.42	[3.20, 8.24]	4.82	[3.19, 7.49]	0.286
**VOC**					
Area sources	130.90	[98.01, 154.1]	129.9	[94.51, 161.3]	0.567
Point sources	2.76	[0.11, 21.92]	0.23	[0.0, 6.21]	<0.000
Mobile sources	32.46	[21.46, 55.62]	31.78	[19.70, 54.00]	0.818
Natural sources	0.53	[0.24, 0.98]	0.44	[0.16, 0.81]	0.065#
Overall emissions	187.60	[132.10, 235.50]	184.10	[131.3, 232.0]	0.664
**TOX**					
Area sources	49.21	[37.91, 61.63]	47.29	[35.38, 64.81]	0.705
Point sources	1.12	[0.04, 8.68]	0.05	[0.0, 3.15]	<0.000
Mobile sources	8.56	[5.438, 13.88]	8.51	[5.18, 13.58]	0.856
Natural sources	0.16	[0.06, 0.27]	0.13	[0.04, 0.26]	0.103
Overall emissions	67.74	[48.25, 89.63]	63.00	[45.92, 81.16]	0.240
**TOC**					
Area sources	161.2	[126.4, 192.7]	158.5	[119.2, 199.4]	0.893
Point sources	3.14	[0.17, 23.95]	0.29	[0.0, 6.82]	<0.000
Mobile sources	35.14	[22.96, 59.27]	34.45	[21.45, 57.25]	0.894
Natural sources	0.53	[0.24, 0.98]	0.44	[0.16, 0.81]	0.065#
Overall emissions	228.6	[165.1, 282.0]	221.60	[158.4, 272.2]	0.538

*IQR* Interquartile Range [25%, 75%].

# Marginally significant.

**Fig. 1:**
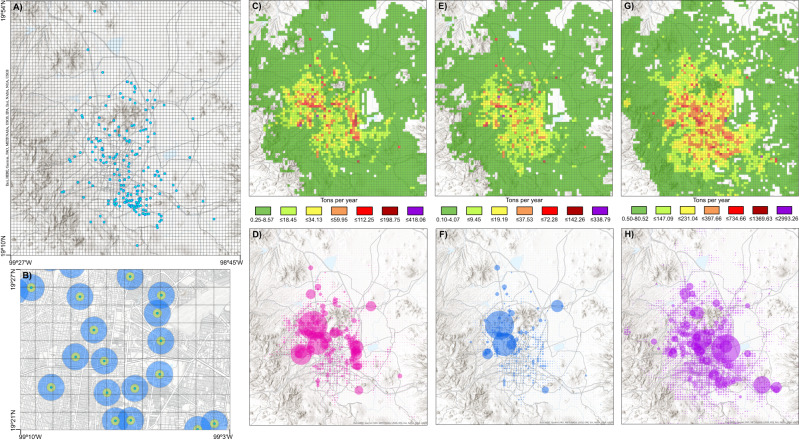
Geographical distribution map of the study population and the levels of atmospheric pollutants in the MCMA through pollutant modeling. **A** Spatial distribution of the study population in the MCMA in the emission modeling grid. **B** Arbitrary location points used as example of two geographical buffers used in the analysis to calculate the exposure for our study population; the 250-m buffer radius is in yellow, and the 750-m buffer radius is in blue. Spatial distribution of annual concentrations of PM10, PM2.5, and VOC (tons per year) in **C**, **E**, and **G**, respectively. The data represent overall emissions as the sum of area, point, mobile, and natural sources for each group of pollutants. Spatial distribution of the annual concentrations of PM10, PM2.5, and VOC in **D**, **F** and **H**, respectively, in proportional symbols.

### Correlations between air pollutant emissions from different sources and DNA adduct levels

Ambient air pollutants were positively and significantly correlated with UCB adducts, but not with MB adducts. Ambient air pollutants in the 750 m radius, but not in the 250 m radius, were positively and significantly correlated with UCB adducts (Table 3). For PM10 (750 m radius), area sources, point sources and overall emissions were positive and significantly correlated with UCB adducts. For PM2.5 (750 m radius), only point sources were positive and significantly correlated with UCB adducts. VOC and TOC from point sources (750 m radius) were positive and significantly correlated with UCB adducts.

**Table 3:** Spearman correlations between the estimated air pollutants in the 750 and 250 m geographical radii, and cord blood and maternal blood adducts.

	Buffers used to estimate the level of exposure (radius around households)					
	750 m			250 m		
Pollutant and source	MB Adducts	UCB Adducts	CMR	MB Adducts	UCB Adducts	CMR
	ρ coefficient	ρ coefficient	ρ coefficient	ρ coefficient	ρ coefficient	ρ coefficient
**PM10**						
Area sources	0.14#	0.17*	0.07	0.06	0.12	0.10
Point sources	0.13	0.19*	0.07	0.01	0.09	0.09
Mobile sources	−0.09	0.09	0.19*	−0.10	0.05	0.16*
Natural sources	−0.23**	−0.16*	0.03	−0.21**	−0.12	0.08
Overall emissions	0.10	0.19*	0.12	0.05	0.11	0.11
**PM2.5**						
Area sources	−0.02	0.08	0.12	−0.08	−0.01	0.10
Point sources	0.12	0.18*	0.07	0.01	0.09	0.09
Mobile sources	−0.06	0.11	0.19*	−0.07	0.08	0.16*
Natural sources	−0.24**	−0.16*	0.04	−0.21**	−0.12	0.08
Overall emissions	0.01	0.14#	0.15*	−0.04	0.05	0.13
**VOC**						
Area sources	−0.07	0.02	0.07	−0.04	−0.03	0.01
Point sources	0.10	0.16*	0.07	0.06	0.11	0.06
Mobile sources	−0.12	0.05	0.19*	−0.13	−0.00	0.13#
Natural sources	−0.23**	−0.15*	0.09	−0.19*	−0.12	0.06
Overall emissions	−0.02	0.08	0.11	−0.04	0.01	0.07
**TOX**						
Area sources	−0.06	0.03	0.08	−0.03	−0.02	0.01
Point sources	−0.02	0.09	0.07	−0.03	0.04	0.05
Mobile sources	−0.07	0.07	0.17*	−0.08	0.02	0.12
Natural sources	−0.23**	−0.14#	0.11	−0.18*	−0.10	0.07
Overall emissions	−0.06	0.06	0.12	−0.06	−0.02	0.03
**TOC**						
Area sources	−0.04	0.03	0.06	−0.02	−0.00	0.01
Point sources	0.08	0.17*	0.08	0.04	0.12	0.07
Mobile sources	−0.13	0.04	0.19*	−0.15*	−0.02	0.13#
Natural sources	−0.23**	−0.15*	0.09	−0.19*	−0.12	0.06
Overall emissions	−0.01	0.08	0.10	−0.02	0.04	0.07

#*P* < 0.06 (Marginal) *P < 0.05, **P < 0.01.

All pollutants from natural sources were negative and significantly correlated with MB adducts in the two radii (250 and 750 m). Ambient air pollutants from natural sources were negatively and significantly correlated with UCB (PM10, PM2.5, VOC and TOC) in the 750 m radius, but not in the 250 m radius (Table 3).

Regarding CMR, PM10 and PM2.5 from mobile sources were significantly and positively correlated with CMR for the two radii, whilst PM2.5 from overall emissions was positive and significantly correlated with CMR for the 750 m radius. In addition, VOC, TOX and TOC emissions from mobile sources were positive and significantly correlated with CMR for the 750 m radius (Table 3).

### Correlations between air pollutant emissions and neonatal intrauterine growth

Correlations between different sources of air pollutants and newborn characteristics are shown in Table 4. PM10 from area sources and overall emissions were significantly negatively correlated with BW, BL, and GA for the two radii (250 and 750 m). In contrast, PM10 emissions from natural or biogenic sources were positively correlated with BW, BL, and GA for the two radii.

**Table 4:** Spearman correlations between air pollutants and birth weight, birth length and gestational age in two geographical radii.

	Buffers used to estimate the level of exposure (radius)					
	750 m			250 m		
Pollutant and source	Weight	Length	Gestational age	Weight	Length	Gestational age
	ρ coefficient	ρ coefficient	ρ coefficient	ρ coefficient	ρ coefficient	ρ coefficient
**PM10**						
Area sources	−0.22***	−0.17**	−0.28****	−0.23***	−0.18**	−0.27****
Point sources	−0.04	−0.13*	−0.14*	−0.00	−0.15*	−0.01
Mobile sources	−0.09	−0.07	−0.10	−0.11	−0.07	−0.15*
Natural sources	0.17*	0.15*	0.22**	0.17**	0.12*	0.20**
Overall emissions	−0.20**	−0.21**	−0.28****	−0.21**	−0.21**	−0.29****
**PM2.5**						
Area sources	−0.12*	−0.09	−0.10	−0.13*	−0.11	−0.12#
Point sources	−0.06	−0.13*	−0.11	−0.02	−0.15*	−0.01
Mobile sources	−0.11#	−0.10	−0.14*	−0.13*	−0.09	−0.19**
Natural sources	0.17**	0.15*	0.22**	0.18**	0.12*	0.20**
Overall emissions	−0.13*	−0.16*	−0.20**	−0.15*	−0.18**	−0.22**
**VOC**						
Area sources	0.03	0.01	0.03	0.03	0.01	−0.02
Point sources	0.05	−0.07	0.02	0.08	−0.10	0.07
Mobile sources	−0.06	−0.05	−0.07	−0.09	−0.05	−0.11
Natural sources	0.19**	0.16*	0.25***	0.19**	0.14*	0.23***
Overall emissions	0.01	−0.04	−0.05	0.00	−0.03	−0.08
**TOX**						
Area sources	0.05	0.02	0.04	0.05	0.04	−0.03
Point sources	0.06	−0.04	0.02	0.02	−0.10	0.06
Mobile sources	−0.07	−0.06	−0.11	−0.11	−0.06	−0.15*
Natural sources	0.19**	0.16*	0.25***	0.20**	0.15*	0.23***
Overall emissions	0.01	−0.01	−0.04	−0.02	−0.02	−0.08
**TOC**						
Area sources	0.04	0.02	0.05	0.01	0.02	−0.02
Point sources	0.06	−0.05	0.02	0.07	−0.10	0.05
Mobile sources	−0.06	−0.05	−0.07	−0.08	−0.05	−0.10
Natural sources	0.19**	0.16*	0.25***	0.20**	0.15*	0.23***
Overall emissions	0.02	−0.02	−0.02	−0.02	−0.02	−0.08

#*P* < 0.06 (Marginal) *P < 0.05, **P < 0.01, ***P < 0.001, ****P < 0.0001.

PM2.5 overall emissions were negatively correlated with BW, BL and GA for the two radii (250 and 750 m). Similar to that observed for PM10, PM2.5 emissions from natural sources were positively correlated with BW, BL, and GA for the two radii. Regarding organic compounds, VOC, TOX and TOC emissions from natural sources were positively correlated with BW, BL, and GA for the two radii. Only TOX from mobile sources (250 m) was negatively and significantly correlated with GA (Table 4).

### Multiple linear regression models to assess associations between PMs, UCB adducts and birth anthropometry

Linear regression models adjusted for potential confounders showed significant associations between PM10 from overall emissions and UCB adducts. For example, a 1% increase in PM10 (tons per year; 750 m radius) was associated with an increase of 0.21% [95% CI: 0.04, 0.38%] in UCB adducts/10^8^ nucleotides. The association between PM2.5 and UCB increase in the 750 m radius did not reach statistical significance (*P* = 0.074). Overall, associations between PM10 and PM2.5 and UCB adducts in the 250 m radius were not statistically significant. On the other hand, PM10 from overall emissions were significantly associated with BW, BL and GA decrease in the two radii. However, BW was the outcome more impacted by PM exposure. For instance, a 1% increase PM10 (tons per year; 750 m radius) was associated with a decrease of 2.12 [95% CI: −3.80, −0.44] g in BW; whilst, a 1% increase in PM10 (tons per year; 250 m radius) was associated with a decrease of 2.35 [95% CI −3.99, −0.70] g in BW. PM2.5 was associated with a decrease in BL and GA in the 750 m radius and BW, BL and GA decrease in the 250 m radius. For example, per 1% increase in PM2.5 (tons per year; 250 m radius), BW decreases 2.01 g [95% CI: −3.99, −0.039] g. Our results showed that PMs, both PM2.5 and PM10 from overall emissions, were negatively associated with newborn intrauterine growth (BW, BL and GA) mainly BW (Table 5).

**Table 5:** Multiple regression analyses to assess associations between PM10 and PM2.5 overall emissions and UCB adducts and birth anthropometry parameters in 250 m and 750 m buffers from household participants.

	Overall emissions PM10		Overall emissions PM2.5	
**750** **m radius**				
Outcomes	Coeff. β [95% CI]	*p*-value	Coeff. β [95% CI]	*p*-value
Log UCB Adducts	0.21 [0.04, 0.38]	**0.013**	0.16 [−0.01, 0.34]	0.074
Birth Weight (g)	−211.9 [−379.8, −44.07]	**0.014**	−141.0 [−328.1, 46.01]	0.139
Birth Length (cm)	−1.07 [−1.83, −0.31]	**0.006**	−0.85 [−1.69, −0.00]	0.050
Gestational Age (weeks)	−0.74 [−1.19, −0.28]	**0.002**	−0.55 [−1.06, −0.04]	**0.034**
**250** **m radius**				
	Coeff. β [95% CI]	*p*-value	Coeff. β [95% CI]	*p*-value
Log UCB Adducts	1.15 [−0.01, 0.31]	0.08	1.11[−0.08, 0.30]	0.263
Birth Weight (g)	−234.5 [−398.7, −70.24]	**0.005**	−201.2 [−398.6, −3.86]	**0.046**
Birth Length (cm)	−1.09 [−1.84, −0.35]	**0.004**	−0.96 [−1.85, −0.06]	**0.036**
Gestational Age (weeks)	−0.79 [−1.23, −0.34]	**0.0006**	−0.70 [−1.24, −0.17]	**0.010**

Overall PM10 and PM2.5 emissions and UCB adducts were log transformed. Models were adjusted for maternal occupation (housewife or employed), maternal age, self-reported environmental tobacco exposure (at least one active smoker living in the same address) and parity.

Bold numbers represent statistical significance (*p* < 0.05).

Additionally, we assessed associations between organic compounds (VOC, TOX and TOC) and DNA adducts and birth anthropometry parameters using linear regression models adjusted for potential confounders. Overall, we found no statistically significant associations (data not shown).

## Discussion

Our results showed higher susceptibility to DNA adduct formation related to ambient air pollutants in newborns than mothers. In models adjusted for potential confounders, we observed associations between PM10 exposure and UCB increase. Regarding birth anthropometry, both PM2.5 and PM10 were associated BW, BL and GA decrease in models adjusted for potential confounders. Overall, we observed no significant associations between VOC, TOX and TOC and UCB adducts or birth anthropometry parameters in models adjusted for potential confounders. Contaminants emitted from natural sources, PMs and organic compounds, were negative correlated with UCB adducts and positive correlated with birth anthropometry parameters. These correlations were no longer significant in models adjusted for potential confounders.

### Differences in ambient air pollutants between 750 m radius and 250 m radius

Overall, PM10, PM2.5,VOC, TOX and TOC concentrations were statistically significantly higher in the 750 m radius than in the 250 m radius only from point sources emissions. These results show the importance of the contribution of the industrial, commercial, or service operations at the periphery (750 m) of household participants. In this study, we found positive and significant correlations between point or area emission sources of contaminants and UCB adducts, suggesting that these emission sources may be a risk factor for newborn health. According to the SEDEMA emissions inventories, area pollutant sources are one of the main sources of pollutants in the MCMA, generating approximately 58% of the total annual emissions of PM10, 47% of the emissions of PM2.5, 64% of VOC emissions, and up to 78% of TOC emissions. On the other hand, point emission sources generate approximately 8% of annual PM10 emissions, 15% of total PM2.5 emissions, and 6% of VOC emissions [8, 9].

### DNA adducts in mothers and newborns participants

Our results showed a positive correlation between MB and UCB adducts, suggesting transplacental transfer of organic compounds to fetal tissues [30, 31]. This result aligns with a previous finding from our group, observing a significant correlation between maternal and fetal DNA adducts [11]. Additionally, we found 60% of the newborns showed higher levels of adducts than their mothers (CMR > 1) suggesting increased fetal susceptibility to DNA adduct formation or less DNA repair activity in the fetus [11, 39, 40].

### Associations between ambient air pollutants from different sources and DNA adducts

PM10 (point and area sources, overall emissions) and PM2.5 (point emissions) in the 750 m radius were significant and positive correlated with UCB adducts, but not MB adducts. VOC and TOC from point sources were positively correlated with UCB adducts.

In multivariate models adjusted for potential confounders, only PM10 (750 m buffer) exposure was associated with an increase in UCB adducts and no associations were observed with MB adducts. These results are in line with previous findings suggesting higher susceptibility in DNA adduct formation related to PM exposure in newborns compared to mothers [11, 30, 39]. PM composition includes PAH, and a study in Mexico City showed that benzo [ghi], perylene and pyrene were the most abundant PAH compounds in PM [32]. PAH are metabolized to form phenolic compounds and reactive epoxides, which can form PAH-DNA adducts [40]. According to our findings, García-Suástegui et al. 2011 [41] and Maciel-Ruiz et al. 2019 [11] reported a relationship between PM10 exposure and PAH-DNA adducts levels in MCMA habitants. The mechanisms related to DNA adduct formation associated with PM might involved inflammation and ROS production [33, 42]. For example, a study with A549 cells exposed to PM showed an increase in PAH-DNA adduct formation related to pro-inflammatory cytokines (IL-6 and IL-8) [42].

### Associations between ambient air pollutants from different sources and birth antropometry parameters

PM10 and PM2.5 from overall emission were correlated with a decrease in birth anthropometry parameters such as BW, BL, and GA in the two geographic radii. Overall, we found no significant and negative correlations between VOC, TOX and TOC emissions and birth anthropometry parameters. Only TOX from mobile sources was negatively and significantly correlated with GA (250 m radius). Our results for organic compounds contrast with previous studies showing the adverse health effects of prenatal exposure to VOC and neonatal characteristics [22, 43].

This study found statistically significant correlations between PMs, VOC, TOX, and TOC emissions from natural sources, a decrease in DNA adducts, and an increase in BW and BL, suggesting a “protective” role of these pollutants from natural sources. However, pollutants from natural sources were no longer significant associated with birth anthropometry parameters or DNA adducts in models adjusted for potential confounders. Pollutants from natural sources comes from patches of vegetation [10, 36] and a lower access to green areas are linked to lower socioeconomic status in Mexico City [44]. Therefore, a beneficial effect of air pollutants from natural sources might be difficult to disentangle due to the high correlation between access to green areas, SES and birth anthropometry parameters as suggested by previous studies [44–50].

In linear regression models adjusted for potential confounders, only PMs were significantly and negatively associated with anthropometry at birth. We found 2.35 g decrease in BW associated with a 1% increase in overall PM10 (250 m radius) and 2.01 g decrease in BW associated with a 1% increase in overall PM2.5 emissions (250 m radius). A meta-analysis conducted by Stieb et al. 2012 using data from 7 epidemiologic studies reported a decrease in BW (pooled estimates) of 16.8 g associated with PM10 (per each 20 μg/m3 increase) exposure for the entire pregnancy [51]. Similarly, in this meta-analysis, PM2.5 exposure (per each 10 μg/m3 increase) for the entire pregnancy was associated with a decrease in BW (pooled estimates) of 23.4 g for the whole pregnancy [51]. Comparing our results with this meta-analysis or other studies is difficult because of differences in exposure method assessment (geographical modelling vs others), time-window period (annual levels vs entire pregnancy) and the magnitude of increase (ton per year vs μg/m3 increase) in PM10 or PM2.5 exposure levels associated with BW, among others. However, despite all these differences, our results align with previous epidemiologic studies showing a decrease in BW associated with prenatal PM exposure [52, 53].

The results of our study should be interpreted considering its strengths and limitations. The strengths of our study include information on different emission sources for PM2.5, PM10, VOC, TOX and TOC around the residence of study participants; DNA adduct levels in maternal and umbilical cord blood as biomarkers of DNA damage; associations between PMs and adduct levels and birth anthropometry parameters using geographical modelling and MEI data. Limitations of our study consist of the lack of information on air pollution exposure at the place of work or during the commute that might impact pollutant exposure levels; we do not have information on indoor air pollution (cooking, household solvents, etc.). Since we have annual exposure levels of contaminants, we were not able to analyze the link between pollutants exposure during periods of susceptibility (e.g. trimester of pregnancy) and DNA adducts and birth parameters. Additionally, we were not able to consider seasonal variation of air pollutants that might impact exposure-outcome associations. We employed the most recent MEI close to the date of delivery of the study participants because when comparing 2014 and 2016 MEI data, we found no significant variations on PM, VOC, TOX and TOC for most of the emission sources (data not shown) and 2015 MEI was not available. Exposure-outcomes associations using annual average of pollutants might biased our results to the null because of the loss of variability in the data [54]. Thus, we might be underestimating adverse health effects of air pollutants. Although, socioeconomic status can also impact our results [50]. We anticipated a similar SES from the study design because participants were recruited from Public Hospitals, where assist patients with similar income. For 102 participants, we had information on income level and 43.1% (44 out of 102) and 35.3% (36 out of 102) reported a maximum monthly income of 6,000 and 9,000 Mexican pesos that corresponded to the III to Vth decile of the 2016 National Survey of Household Income and Expenditure reported by INEGI ((http://en.www.inegi.org.mx/programas/enigh/nc/2016/). Finally, we excluded from our study preterm births and active smokers; thus, our results might be subjected to selection bias due to the inclusion of specific group of women [55]. Moreover, our study is subjected to exposure-health effects bias because exposure is related to prespecified eligibility criteria (PM exposure and preterm birth) [56]. Besides all these limitations, our study showed adverse health effects related to PMs and organic compounds in pregnant women of MCMA using a geographical modelling approach.

## Conclusions

Prenatal exposure to particulate matter (PM) from overall emissions is associated with newborn’s DNA adduct formation and birth weight and length decrease. Our results also suggested that organic compounds (VOC, TOC and TOX) from any emission sources are not associated with newborns’ DNA adduct formation or birth anthropometry parameters. Our results highlight the need to reduce environmental exposure to PMs from overall emissions in susceptible periods of life.

## Supplementary information

Supplementary Material

## Data Availability

Per Instituto de Investigaciones Biomédicas, IMSS and CimiGen Institutional Review Board approval, the data that support the findings of this study are restricted for transmission to those outside the primary investigative team. Data sharing with investigators outside the team requires IRB approval. Requests may be submitted to Maria E. Gonsebatt (margeng@unam.mx).
