# Supplementary material for: Geographical approach analysis of the impact of air pollution on newborn intrauterine growth and cord blood DNA damage in Mexico City
Source: J Expo Sci Environ Epidemiol. 2023 Dec 12;34(5):907–16. doi: 10.1038/s41370-023-00618-x (PMC11446826; doi:10.1038/s41370-023-00618-x)
Supplement: Supplementary file 1 — Supplementary Material [file 41370_2023_618_MOESM1_ESM.docx]

**Supplementary Material**

**Supplemental Table 1.** Inventory of VOC (volatile organic compounds), TOC (total organic compounds) and TOX (toxic organic compounds) emissions monitored in the MCMA [8,9].

| **VOC** | **TOC** | **TOX** |
| --- | --- | --- |
| Toluene | Carbonates | Toluene |
| Xilene isomers | Metallic carbides | Xilene isomers |
| 1,1,1, Trichloroethene | Carbon monoxide | 1,1,1, Trichloroethene |
| Isopropyl alcohol | Carbonic acid | Isopropyl alcohol |
| Methanol | Carbon dioxide | Methanol |
| Trichloro trifluoroethane | Methane | Trichloro trifluoroethane |
| Trichloroethylene | Ethane | Trichloroethylene |
| Hexane | Acetone | Hexane |
| Dimethylamine | 1,1,1, Trichloroethene | Dimethylamine |
| Dichloromethane | Chlorofluorocarbons | Dichloromethane |
| Ethylbencene | Hydrochlorofluorocarbons | Ethylbencene |
| Methyl Tert-Butyl Ether | Hydrofluorocarbons | Methyl Tert-Butyl Ether |
| Perchloroethylene | Perfluorocarbons | Perchloroethylene |
| Benzene | Dichloromethane | Benzene |
| Methyl Bromide | Perchloroethylene | Methyl Bromide |
|  | Benzene | Other (including metals) |
|  | Xylene |  |
|  | Toluene |  |
|  | Other hydrocarbons |  |
|  | Aldehydes |  |
|  |  |  |
